# The association of sleep difficulties with health-related quality of life among patients with fibromyalgia

**DOI:** 10.1186/1471-2474-13-199

**Published:** 2012-10-17

**Authors:** Jan-Samuel Wagner, Marco D DiBonaventura, Arthi B Chandran, Joseph C Cappelleri

**Affiliations:** 1 Health Outcomes Practice, Kantar Health, 11 Madison Avenue, 12th Floor, New York, NY, 10010, USA; 2 Pfizer Inc., 235-09-02, 235 East 42nd Street, New York, NY, 10017, USA; 3 Pfizer Inc., 445 Eastern Point Road, MS 8260-2502, Groton, CT 06340, USA

**Keywords:** Fibromyalgia, Sleep, Insomnia, Health-related quality of life, Pain

## Abstract

**Background:**

Difficulty sleeping is common among patients with fibromyalgia (FM); however, its impact on health-related quality of life (HRQoL) is not well understood. The aim of the current study was to assess the burden of sleep difficulty symptoms on HRQoL among patients with FM.

**Methods:**

The current study included data from the 2009 National Health and Wellness Survey (N=75,000), which is a cross-sectional, Internet-based survey representative of the adult US population. The prevalence of sleep difficulty symptoms among patients with FM (n=2,196) were compared with matched controls (n=2,194), identified using propensity-score matching. Additionally, the relationship between the number of sleep difficulty symptoms (none, one, or two or more) and HRQoL (using the SF-12v2) was assessed using regression modeling, controlling for demographic and health history variables.

**Results:**

Of the 2,196 patients with FM, 11.2% reported no sleep difficulty symptoms, 25.7% reported one sleep difficulty symptom, and 63.05% reported two or more sleep difficulty symptoms. The prevalence of sleep difficulty symptoms was significantly higher than matched controls. Patients with one and two sleep difficulty symptoms both reported significantly worse HRQoL summary and domain scores relative to those with no sleep difficulty symptoms (all p<.05). Further, the relationship between sleep difficulty symptoms and HRQoL was significantly different between those with FM than matched controls, suggesting a uniqueness of the burden of sleep difficulties within the FM population.

**Conclusions:**

Among the FM population, sleep difficulty symptoms were independently associated with clinically-meaningful decrements in mental and physical HRQoL. These results suggest that greater emphasis in the treatment of sleep difficulty symptoms among the FM population may be warranted.

## Background

Fibromyalgia (FM) is a chronic disorder characterized by widespread pain of the muscle and connective tissues, and pain in response to touch or pressure [1]. Often accompanied by non-specific symptoms, such as fatigue, depressive mood, and sleep difficulties [2], FM affects approximately 5 million Americans [3]. As it largely affects a working-age population, and is associated with increased resource use and disability, FM is responsible for substantial societal costs. Indeed, prior research on managed care patients with FM found an average of $10,911 (standard deviation = $16,860) in healthcare expenses per patient per year during 2001–2004 [4]. Moreover, patients with FM reported short-term disability at a greater rate than patients with rheumatoid arthritis (20% vs. 15% reported any short-term leave) [4]. Costs also increase with severity, with patients with severe symptoms reporting more than three times the costs of patients with mild symptoms [5].

The societal impact of FM is not limited to economic costs, however. Patients report that FM symptoms substantially impact their quality of life by disrupting relationships, causing social isolation, reducing productivity in activities of daily living, and complicating physical activity [6]. In a recent review of 37 studies, Hoffman & Dukes found patients with FM report mental health-related quality of life (HRQoL) scores 1 standard deviation below the United States (US) population mean and physical HRQoL scores 2 standard deviations below the US population mean [7]. In fact, HRQoL among patients with FM have been found to be similar to or worse than patients with rheumatoid arthritis [8] and other pain conditions [7].

Patients with FM have been found to be significantly more likely to experience difficulties initiating or maintaining sleep than controls (OR=4.56, 95% CI: 4.10-5.06) [9]. In particular, previous studies have identified difficulty falling asleep, staying asleep and waking up too early in the morning as the most common sleep-related symptoms among the FM population [9-12]. Such sleep difficulties have been associated with negative affect and mood, and pain, which, in turn, have been associated with decrements in physical functioning [13-16]. Moreover, in qualitative interviews, patients with FM have reported that sleep disturbances substantially affect their quality of life [5,17]. However, few studies have assessed the direct association of sleep difficulties with decrements in HRQoL among this patient population.

There were several aims of the current study. One aim was to determine the prevalence of sleep difficulty symptoms among those with FM in comparison with those without FM. The second aim was to examine which demographic and health history variables were significantly associated with the presence of these sleep difficulty symptoms. Lastly, the third aim was to examine the relationship between these sleep difficulty symptoms and HRQoL among patients with FM and determine whether these relationships differed from a non-FM control sample.

## Methods

### Data source

Data were obtained from the 2009 wave (*N* = 75,000) of the US National Health and Wellness Survey. The NHWS is an annual, cross-sectional, Internet-based survey administered to a sample of adults (18 years and older) identified through a web-based panel. Members of the panel are recruited through emails, Internet newsletter campaigns, website banner placements, and registration with panel partners. All panel members agreed to become panel members and registered through unique email addresses. Of 501,239 persons contacted to participate in the 2009 NHWS, 92,759 responded (an 18.5% response rate). Of those who responded, 75,000 gave their informed consent, met the inclusion criteria (aged 18 or over), and completed the survey instrument. To mimic the demographic composition of the US general population, a stratified random sampling procedure was implemented when recruiting participants for the NHWS. The NHWS sample, US census, and other national surveys have been compared elsewhere [18]. Institutional review board approval for the 2009 US NHWS was granted by Essex IRB (Lebanon, NJ).

### Sample

All respondents to the 2009 US NHWS were included in the analysis (N=75,000).

### Measures

#### Sleep difficulties

Although no sleep scale was included in the NHWS, all respondents were asked whether they experienced difficulty falling asleep, difficulty staying asleep, or waking up too early (the response options for each item were either yes or no). These items, which have been shown to be the most common characteristics of sleep difficulties among patients with FM [9-12], were used to operationally define the presence of sleep difficulties. Severity of sleep difficulties was established based on the number of sleep difficulty symptoms reported (see Table 1). Similar operationalizations have been used elsewhere [9,19]. The primary independent variable was a three-level mutually exclusive group variable: no sleep difficulty symptoms, one sleep difficulty symptom, and two or more sleep difficulty symptoms.

**Table 1:** Criteria for establishing the presence of sleep difficulties in patients

**No sleep difficulty symptoms**	**One sleep difficulty symptom**	**Two or more sleep difficulty symptoms**
Reporting none of the following:	Reporting one of the following:	Reporting two or more of the following:
· Difficulty falling asleep	· Difficulty falling asleep	· Difficulty falling asleep
· Difficulty staying asleep	· Difficulty staying asleep	· Difficulty staying asleep
· Waking too early	· Waking too early	· Waking too early
· Insomnia		
· Sleep difficulties (any)		

#### FM

All respondents in the NHWS were presented with a list of medical conditions and asked to select which ones they had ever experienced (“which of the following conditions have you ever experienced?”). FM (presented as “fibromyalgia” to respondents) was included in this list. All respondents who selected FM were subsequently asked “has your fibromyalgia been diagnosed by a physician?”, with yes/no response options. Only respondents who reported a diagnosis of FM were considered to have FM for the purposes of this study and the remaining respondents were considered controls.

#### Demographic and health history variables

Age, race/ethnicity (non-Hispanic white, non-Hispanic black, Hispanic, or other race/ethnicity), marital status (married/living with partner versus all else), education (high school graduate or less versus some college or higher), annual household income ($25K or less, $25K to <$50K, $50K to <$75K, $75K or more, or decline to answer), employment (full-time, part-time, self-employed, not employed and not looking for work, not employed but looking for work, on disability, retired, student, or homemaker), health insurance (yes versus no), exercise (no days with 20 minutes or more of exercise in the past month, 1–9 days, or 10 or more days), smoking habits (current smoker versus non-smoker), alcohol consumption (consume alcohol versus abstain from alcohol), body mass index (BMI; underweight, normal weight, overweight, obese, or decline to provide weight), and comorbidity burden (using the Charlson Comorbidity Index; CCI [20]) were assessed.

Pain severity (no pain, mild pain, moderate pain, and severe pain) and frequency (no pain, daily pain, 4–6 days of pain per week, 2–3 days of pain per week, weekly pain, 2–3 days of pain per month, 1 day of pain a month or less) information were also measured for each respondent.

#### Health-related quality of life

HRQoL was assessed using the the Medical Outcomes Study Short Form-12 questionnaire (SF-12), version 2, which has a four-week recall period [21]. The SF-12 was developed to replicate the mental (MCS) and physical component summary (PCS) scores and domain scores of the SF-36. The eight health domains include: physical functioning, physical role limitations, emotional role limitations, bodily pain, general health, vitality, social functioning, and mental health. The PCS and MCS are normed to the US population (Mean = 50, SD = 10), with higher scores indicating greater quality of life. Differences of 3 points in MCS and PCS were considered clinically meaningful, in accordance with previous findings [22].

### Analyses

There were four components to the analysis. In the first, the prevalence of sleep difficulty symptoms among patients with FM were reported and compared with those without FM (both unmatched and matched controls) using chi-square tests. Unmatched controls were defined as those who did not report a diagnosis of FM. Matched controls were created using a propensity score matching method [23].

A logistic model was conducted using age, gender, race/ethnicity, and comorbidities (using the CCI) to predict a diagnosis of FM. Propensity score values were saved from this regression and used in a greedy-matching algorithm to assign each patient diagnosed with FM with a single control, whose propensity score was identical. Although there were 2,196 patients diagnosed with FM, only 2,194 patients were able to be matched (the remaining two patients had a pattern of covariates which was too unique to find a suitable control). Therefore, there were 2,194 matched controls.

The second component of the analysis compared the different symptom groups (no sleep difficulty symptoms versus one sleep difficulty symptom versus two or more sleep difficulty symptoms) among those with FM with respect to demographics and health history variables noted above. Comparisons were made with chi-square tests and one-way ANOVAs.

The third component of the analysis consisted of regressing summary and domain scores of the SF-12 onto the number of sleep difficulty symptoms (none, one, or two or more) among patients with FM. These models were conducted using linear regression for complex survey designs (PROC SURVEYREG in SAS v9.1). For the sake of parsimony, only covariates which differed among the groups were included in the model: age, smoking status (non-smoker served as the reference category), pain severity (no pain served as the reference category), and pain frequency (no pain served as the reference category).

The fourth component of the analysis was a comparison of the relationship of sleep difficulty symptoms and HRQoL between those with FM and matched controls. This was accomplished by entering the case variable (diagnosed FM versus matched control), number of sleep symptoms (none, one, and two or more), and the interaction term into a linear regression model (PROC SURVEYREG) for each summary and domain score. Effects coding was used for all variables. This model tested the main effect (i.e., whether HRQoL was different between those with FM and matched controls) as well as the interaction (i.e., whether the relationship between the number of sleep symptoms and HRQoL differed between patients with FM and matched controls).

All analyses applied sampling weights from the NHWS. Although raw sample sizes are provided in many cases, all other statistical information (unless otherwise specified) were weighted to project to the population. Analyses were conducted using SAS v9.1 (Cary, NC) and statistical significance was set at p<.05.

## Results

### Frequency of sleep difficulty symptoms

A total of 2,196 patients in the NHWS reported diagnosis of FM (2.81%). Of these, 269 (11.2%) reported no sleep difficulty symptoms, 574 (25.7%) reported one sleep difficulty symptom, and 1353 (63.05%) reported two or more sleep difficulty symptoms. The prevalence of sleep difficulty symptoms was significantly higher when compared with those without FM (none: 40.7%; one: 29.0%; two or more: 30.3%; p<.0001). Indeed, even when comparing patients with FM with matched controls (those matched on age, sex, ethnicity, and comorbidities), the prevalence of sleep difficulty symptoms was significantly higher (none: 11.2% vs. 32.9% for FM and matched control patients, respectively; one: 25.7% vs. 28.22%, respectively; two or more: 63.1% vs. 38.9%, respectively; p<.0001).

### Demographic and health history comparisons

Among patients with FM, few demographic and health history variables were related to the number of sleep symptoms experienced (see Table 2). Patients with FM who reported sleep difficulties were younger, less likely to be retired and possess health insurance, and more likely to be on disability and to currently smoke. Levels of severe pain increased concomitantly with the number of sleep symptoms (19.08% vs. 30.77% vs. 43.26% for those with none, one, and two sleep difficulty symptoms, respectively). Similarly, reports of daily pain also increased along with the number of sleep difficulty symptoms (49.21% vs. 59.67% vs. 70.88%).

**Table 2:** Demographic and health history differences associated with the number of sleep difficulty symptoms among patients with fibromyalgia

	**No sleep symptoms (n=269)**				**One sleep symptom (n=574)**				**Two or more sleep symptoms (n=1353)**				
**Variable**	**n**	**Weighted n**	**Weighted %**	**SE**	**n**	**Weighted n**	**Weighted %**	**SE**	**n**	**Weighted n**	**Weighted %**	**SE**	**p**
Male	42	100791	14.27%	2.24%	109	291061	17.96%	1.71%	204	595728	15.01%	1.08%	0.2813
Race/ethnicity													0.0701
Non-Hispanic white	231	579865	82.11%	2.72%	480	1297016	80.03%	1.93%	1096	3060560	77.12%	1.34%	
Non-Hispanic black	16	44654	6.32%	1.55%	36	95814	5.91%	0.97%	80	207978	5.24%	0.58%	
Hispanic	12	51952	7.36%	2.08%	34	160905	9.93%	1.63%	82	396647	9.99%	1.06%	
Other	10	29762	4.21%	1.34%	24	66882	4.13%	0.86%	95	303503	7.65%	0.86%	
Greater than high school education	205	548338	77.64%	2.86%	445	1254870	77.43%	1.89%	1041	3115084	78.49%	1.17%	0.8776
Annual household income													0.0906
<$25K	67	164262	23.26%	2.91%	157	453920	28.01%	2.06%	405	1194941	30.11%	1.35%	
$25K to <$50K	75	209604	29.68%	3.2%	162	450214	27.78%	2.04%	431	1254027	31.60%	1.37%	
$50K to <$75K	59	161650	22.89%	2.87%	125	329591	20.34%	1.75%	234	691354	17.42%	1.08%	
$75K or more	47	128776	18.23%	2.65%	100	294962	18.20%	1.74%	228	687512	17.32%	1.1%	
Decline to answer	21	41942	5.94%	1.4%	30	91929	5.67%	1.09%	55	140853	3.55%	0.51%	
Employment													<.0001
Full-time	40	129881	18.39%	2.69%	96	302795	18.68%	1.78%	204	637364	16.06%	1.06%	
Part-time	21	56486	8.00%	1.76%	58	161415	9.96%	1.31%	132	379165	9.55%	0.83%	
Self-employed	17	46951	6.65%	1.62%	30	85771	5.29%	0.99%	81	248640	6.27%	0.69%	
Unemployed, looking for work	12	32426	4.59%	1.35%	22	59646	3.68%	0.8%	75	231083	5.82%	0.68%	
Unemployed, not looking	2	4421	0.63%	0.48%	15	43845	2.71%	0.7%	63	186145	4.69%	0.59%	
Retired	114	258239	36.57%	3.39%	160	391288	24.14%	2.02%	283	715123	18.02%	1.22%	
Disability	40	116650	16.52%	2.46%	133	394200	24.32%	1.89%	379	1154974	29.10%	1.31%	
Student	1	2271	0.32%	0.32%	6	21302	1.31%	0.56%	15	47148	1.19%	0.31%	
Homemaker	22	58909	8.34%	1.81%	54	160354	9.89%	1.34%	121	369046	9.30%	0.83%	
Married/living with partner	162	420899	59.60%	3.41%	353	968068	59.73%	2.26%	816	2426243	61.13%	1.43%	0.8299
Possess health insurance	252	651206	92.21%	1.84%	517	1448371	89.37%	1.38%	1181	3433135	86.51%	0.98%	0.0153
Exercise behavior													0.0624
None in past month	118	297414	42.11%	3.38%	265	709114	43.76%	2.24%	674	1954071	49.24%	1.46%	
1-9 days exercise in past month	59	167526	23.72%	2.97%	157	456390	28.16%	2.05%	306	938221	23.64%	1.29%	
10+ days exercise in past month	92	241295	34.17%	3.25%	152	455112	28.08%	2.06%	373	1076395	27.12%	1.28%	
Alcohol use	140	367023	51.97%	3.44%	299	835869	51.58%	2.27%	672	1991970	50.19%	1.46%	0.8168
Current smoker	58	163896	23.21%	2.95%	171	515809	31.83%	2.11%	405	1227589	30.93%	1.34%	0.0405
Body mass index													0.1211
Underweight	5	9180	1.30%	0.67%	11	35633	2.20%	0.68%	16	42536	1.07%	0.28%	
Normal	53	144538	20.47%	2.87%	128	397673	24.54%	2.01%	277	811375	20.44%	1.17%	
Overweight	80	208488	29.52%	3.13%	168	462626	28.55%	2.04%	346	1061443	26.75%	1.34%	
Obese	120	309173	43.78%	3.39%	257	696615	42.98%	2.23%	682	1956748	49.30%	1.46%	
Decline to answer weight	11	34856	4.94%	1.6%	10	28070	1.73%	0.55%	32	96584	2.43%	0.44%	
Pain severity													<.0001
No pain	79	217295	30.77%	3.16%	129	373430	23.04%	1.94%	217	611959	15.42%	1.03%	
Mild pain	10	32456	4.60%	1.7%	34	87366	5.39%	0.97%	29	83328	2.10%	0.41%	
Moderate pain	113	295400	41.83%	3.42%	217	593646	36.63%	2.16%	514	1503698	37.89%	1.42%	
Severe pain	57	134783	19.08%	2.5%	175	498641	30.77%	2.08%	574	1716877	43.26%	1.46%	
Missing severity	10	26300	3.72%	1.23%	19	67533	4.17%	1%	19	52824	1.33%	0.31%	
Pain frequency													<.0001
No pain	79	217295	30.77%	3.16%	129	373430	23.04%	1.94%	217	611959	15.42%	1.03%	
Daily pain	140	347542	49.21%	3.43%	347	966989	59.67%	2.23%	955	2813110	70.88%	1.31%	
4-6 days per week	18	44232	6.26%	1.55%	40	107597	6.64%	1.07%	105	320622	8.08%	0.8%	
2-3 days per week	11	37374	5.29%	1.9%	28	74165	4.58%	0.91%	43	128377	3.23%	0.51%	
Weekly pain	2	6227	0.88%	0.64%	6	19050	1.18%	0.48%	7	19631	0.49%	0.19%	
2-3 days per month or less	9	27264	3.86%	1.45%	5	11852	0.73%	0.35%	7	22164	0.56%	0.21%	
Missing frequency	10	26300	3.72%	1.23%	19	67533	4.17%	1%	19	52824	1.33%	0.31%	
		**Mean**	**Weighted Mean**	**SE**		**Mean**	**Weighted Mean**	**SE**		**Mean**	**Weighted Mean**	**SE**	**p**
Age		57.56	56.04	1.07		53.36	51.90	0.7		52.47	51.63	0.39	0.0006
Charlson comorbidity index		1.1	1.11	0.11		1.3	1.34	0.13		1.18	1.17	0.05	0.3475

### Association of sleep difficulty symptoms and health-related quality of life

The distributions of all HRQoL summary and domain scores for those with FM are presented in Table 3. Linear regressions were conducted to determine the relationship between the number of sleep difficulty symptoms and HRQoL among those with FM (see Table 4). For all summary and domain scores, the experience of sleep difficulty symptoms was associated with a significant decrement relative to those without any sleep difficulty symptoms. In the case of the normed mental and physical component summary scores, the effects of both one (b’s: -2.79 and −2.42, respectively) and two sleep difficulty symptoms (b’s: -3.91 and −2.84, respectively) approached or exceeded clinically-relevant thresholds (i.e., 3 points).

**Table 3:** Descriptive statistics of summary and domain scores of the SF-12 among patients with fibromyalgia

	**Min**	**% at Min**	**Q1**	**Median**	**Mean**	**Q3**	**Max**	**% at Max**
***Summary scores***								
Mental component summary	3.57	0.05%	31.98	41.07	41.49	51.22	71.53	0.05%
Physical component summary	7.25	0.05%	23.99	31.29	31.29	39.88	63.16	0.05%
Health utilities	0.35	1.50%	0.49	0.57	0.57	0.64	1.00	0.23%
***Domain scores***								
Bodily pain*	0.00	20.49%	25.00	25.00	25.00	50.00	100.00	2.41%
General health*	0.00	13.34%	25.00	25.00	25.00	60.00	100.00	1.55%
Vitality*	0.00	30.78%	0.00	25.00	25.00	50.00	100.00	1.14%
Social functioning*	0.00	12.66%	25.00	50.00	50.00	75.00	100.00	13.93%
Mental health**	0.00	2.73%	37.50	50.00	50.00	75.00	100.00	1.82%
Emotional role limitations**	0.00	8.24%	37.50	50.00	50.00	87.50	100.00	21.36%
Physical role limitations**	0.00	21.04%	12.50	25.00	25.00	50.00	100.00	4.74%
Physical functioning*	0.00	29.96%	0.00	25.00	25.00	50.00	100.00	10.75%

*Note these domains scores from the SF-12 are based only on a five-point range (0, 25, 50, 75, 100).

**Note these domains scores from the SF-12 are based only on a 9-point range (0, 12.5, 25, 37.5, 50, 62.5, 75, 87.5, 100).

**Table 4:** Regression estimates for the number of sleep difficulty symptoms when predicting health-related quality of life

**Dependent variable**		**No symptoms (reference)**	**One symptom**	**Two or more symptoms**
Mental component summary	b	--	−2.786	−3.905
	95% CL	--	(−4.448, -1.124)	(−5.461, -2.35)
	R^2^		0.174	
Physical component summary	b	--	−2.415	−2.835
	95% CL	--	(−4.144, -0.686)	(−4.467, -1.202)
	R^2^		0.241	
Health utilities	b	--	−0.041	−0.054
	95% CL	--	(−0.06, -0.022)	(−0.072, -0.035)
	R^2^		0.284	
Bodily pain	b	--	−5.770	−8.555
	95% CL	--	(−9.729, -1.811)	(−12.239, -4.87)
	R^2^		0.312	
General health	b	--	−6.349	−8.441
	95% CL	--	(−10.358, -2.34)	(−12.222, -4.66)
Vitality	R^2^		0.153	
	b	--	−5.943	−6.637
	95% CL	--	(−9.994, -1.893)	(−10.447, -2.827)
	R^2^		0.097	
Social functioning	b	--	−7.327	−10.838
	95% CL	--	(−11.741, -2.913)	(−14.906, -6.771)
	R^2^		0.205	
Mental health	b	--	−4.366	−8.658
	95% CL	--	(−7.71, -1.021)	(−11.805, -5.511)
	R^2^		0.184	
Emotional role limitations	b	--	−8.094	−7.546
	95% CL	--	(−12.802, -3.387)	(−11.847, -3.245)
	R^2^		0.120	
Physical role limitations	b	--	−9.485	−10.416
	95% CL	--	(−14.043, -4.928)	(−14.683, -6.148)
	R^2^		0.194	
Physical functioning	b	--	−6.015	−6.915
	95% CL	--	(−11.315, -0.715)	(−11.867, -1.963)
	R^2^		0.177	

*Regression coefficients represent the difference in HRQoL for those with one and two symptoms relative to those without any (e.g., those with one symptom and two or more symptoms reported mental component summary scores 2.786 and 3.905 points less, respectively, than those without symptoms). All models controlled for age, smoking status, pain severity, and pain frequency*.

Similarly, decrements in health utility values (b = −0.04 and −0.05 for one and two sleep difficulty symptoms, respectively) were also above clinically-relevant thresholds (i.e., 0.03 points). The SF-12 domain scores for those with one sleep difficulty symptom were equally affected (generally speaking) compared with those with no sleep difficulty symptoms (b’s ranged from −4.37 to −9.49); similarly, the SF-12 domain scores for those with two sleep difficulty symptoms were also equally affected compared with those with no sleep difficulty symptoms (b’s ranged from −6.64 to −10.84).

Additional models were then conducted to determine whether the relationship between sleep difficulty symptoms and HRQoL was different among those with FM than a similar cohort without FM. When compared with matched controls, patients with FM reported significantly worse HRQoL across all summary and domain scores of the SF-12 (i.e., “main effects”; see Table 5; all p<.0001).

**Table 5:** Mean levels of health-related quality of life for patients with fibromyalgia and matched controls as a function of sleep difficulty symptoms

	**Matched control**			**Patients with FM**				
	**No symptoms (n=717)**	**One symptom (n=636)**	**Two or more symptoms (n=841)**	**No symptoms (n=269)**	**One symptom (n=572)**	**Two or more symptoms (n=1353)**	**p (Main effect)**	**p (Interaction)**
Mental component summary	51.20	47.65	43.51	47.26	42.34	40.00	<.0001	<.0001
Physical component summary	46.56	43.51	41.68	35.91	33.24	31.57	<.0001	0.0445
Health utilities	0.77	0.71	0.66	0.64	0.59	0.55	<.0001	<.0001
Bodily pain	75.45	66.86	59.69	46.00	37.94	31.04	<.0001	0.0028
General health	67.34	59.82	54.99	52.86	43.75	39.49	<.0001	0.0097
Vitality	53.66	46.74	37.57	36.52	28.54	25.67	<.0001	<.0001
Social functioning	83.05	73.86	63.59	63.66	52.01	44.92	<.0001	0.0003
Mental health	74.01	64.94	56.79	64.73	55.75	49.06	<.0001	<.0001
Emotional role limitations	83.26	76.47	69.59	69.80	58.26	55.14	<.0001	0.0545
Physical role limitations	73.34	63.38	56.45	48.19	36.39	31.88	<.0001	0.0069
Physical functioning	74.23	64.98	58.95	46.47	39.51	34.59	<.0001	0.0151

The p-value for the main effect represents the significance of the HRQoL differences between patients with FM and matched controls across all levels of symptoms. The p-value for the interaction represents the significance of whether the change from no symptoms to one symptom to two or more symptoms was different between patients with FM and matched controls.

However, the relationships between the number of sleep difficulty symptoms and HRQoL was significantly stronger among those with FM. In other words, significant interactions were observed for all summary and domain scores (with the exception of emotional role limitations). In most cases, the decrement between no sleep difficulty symptoms and one sleep difficulty symptom was larger among those patients with FM; conversely, the quality of life decrement between one and two or more sleep difficulty symptoms was larger among matched controls.

## Discussion

The objective of the current study was to assess the impact of sleep difficulties on HRQoL among patients with FM. Despite the research on the sleep-related symptoms experienced by those with FM, no study to our knowledge has examined the relationship of these symptoms with HRQoL, especially in comparison with a matched control group. Our results suggest sleep difficulties are pervasive among the FM population, as over 88% of patients reported some level of sleep difficulties, as defined by experiencing either difficulty falling asleep, difficulty staying asleep, or waking up too early. Nearly 63% of patients with FM reported experiencing at least two of the above symptoms. These figures were significantly higher than those without FM, even those matched with FM patients.

Replicating past literature, our sample of patients with FM reported significant decrements in both MCS (41.49) and PCS (31.29) scores relative to population norms (50 and 50, respectively). Indeed, the one and two standard deviation differences, respectively, in MCS and PCS compared with the population mean is nearly identical to the results reported by previous literature [6,7]. On an absolute level, these levels of HRQoL are worse than reported in the same survey for patients with severe osteoarthritis, chronic obstructive pulmonary disease, atrial fibrillation, hepatitis C, arthritis, and back pain, among others [24-28], However, our findings suggest that the presence of sleep difficulties poses an additional burden on patients with FM. Our results suggest that sleep difficulty symptoms has an independent, and significant, clinically-meaningful effect on HRQoL among the FM population. Past research has suggested a three-point between-groups difference in MCS and PCS is often associated with a clinically-meaningful difference [21]. The comparison between one sleep difficulty symptom and no sleep difficulty symptoms approached this threshold while the comparison between two sleep difficulty symptoms and no sleep difficulty symptoms exceeded it, even after adjusting for confounding variables.

It is particularly important to note that these models controlled for pain severity and frequency. Naturally, severe pain and frequent pain (as confirmed in Table 2) would be expected to have a significant effect on sleep symptoms, as also demonstrated in prior research [14,16,20,22]. Yet, even accounting for the higher prevalence of pain severity and frequency among those with more sleep difficulty symptoms, worse HRQoL summary and domain scores were observed. This suggests that sleep difficulties have an independent effect on HRQoL among those with FM, beyond any potential effect of the pain experience.

Also noteworthy was that the relationship between sleep difficulties and HRQoL varied between those with FM and matched controls. In many cases, the introduction of a single sleep difficulty symptom was associated with a larger decrement in HRQoL among patients with FM, however, the introduction in a second sleep difficulty symptom was associated with larger decrement in HRQoL among matched controls. Further research may be necessary to ascertain the cause of the discrepancy. One possibility is that sleep difficulties are generally more burdensome for patients with FM, however, given the nature of the disease, a floor effect is reached upon the introduction of the second sleep difficulty symptom. In other words, patients with FM are so burdened already by their condition that the introduction of an additional sleep difficulty symptom does not affect their HRQoL as much as it would a patient without FM. Regardless, our preliminary evidence suggests that the pattern of the relationship between sleep difficulty symptoms and HRQoL is unique among those with FM.

The effect of sleep difficulty symptoms extended beyond HRQoL. In unadjusted comparisons, patients who reported sleep difficulties showed higher rates of disability than those without sleep difficulties. Although beyond the scope of the present analysis, the effect of sleep difficulties on participation in the labor force and productivity at work may also need to be considered in future research.

In sum, the results suggest patients with FM experience considerable difficulty initiating and maintaining sleep. The presence of these sleep difficulty symptoms have a significant and clinically-meaningful impact on HRQoL, even after accounting for a range of confounding variables. The study results suggest the improved management of these sleep difficulty symptoms among patients with FM may lead to clinically-relevant improvements in HRQoL. The alleviation of pain could have an important effect of improving sleep, but more research would be necessary to establish this causal pathway. Indeed, the relationship between pain and sleep does appear bidirectional [29]. Of course, since the effect of sleep difficulty symptoms on HRQoL was observed even after controlling for pain, the management of sleep difficulties likely extends beyond the mere alleviation of pain.

### Limitations

Several limitations should be noted from the results of this study. Given the cross-sectional design of the study, the causal inference cannot be determined. Although alternative explanations have been included (such as comorbidities and, demographic confounders), it is possible other unmeasured variables might explain the relationship between sleep difficulties and HRQoL. Because of the self-reported nature, recall bias may have introduced additional error into the observed associations. As described before, the sleep difficulty groups were not defined by a sleep scale but rather using three symptoms of initiating and maintaining sleep to operationalize sleep difficulty severity. It should also be emphasized that although the NHWS is demographically representative of the US population, the sample in the current study of FM patients may differ with respect to healthcare attitudes or healthcare engagement (among other variables) that could affect the size and direction of the relationships observed here.

## Conclusions

Sleep difficulties were found to have significant and clinically meaningful deleterious effects on HRQoL among the FM population. Effective treatment of sleep difficulties may improve HRQoL among the FM population.

## Abbreviations

FM: Fibromyalgia; SD: Standard deviation; HRQoL: Health-related quality of life; US: United States; NHWS: National Health and Wellness Survey; IRB: Institutional review board; BMI: Body mass index; CCI: Charlson comorbidity index; SF: Short Form; MCS: Mental component summary; PCS: Physical component summary.

## Competing interests

This study was conducted by Kantar Health on behalf of Pfizer Inc, which funded the study. JSW and MD are full-time employees of Kantar Health who were paid consultants to Pfizer in connection with the analysis and the development of this manuscript. AC and JCC are full-time employees of Pfizer, Inc.

## Authors’ contributions

JSW participated in the design, coordination, and analysis of the study, and drafted the manuscript. MD was engaged in the conception and design of the study and participated in the manuscript’s drafting and editing. AC was involved in the conception, design, and coordination, of the study, and critically evaluated and provided input to the manuscript. JCC was involved in the design of the study, provided oversight on the analyses, critically evaluated and provided input to the manuscript. All authors read and approved the final manuscript.

## Pre-publication history

The pre-publication history for this paper can be accessed here:

http://www.biomedcentral.com/1471-2474/13/199/prepub
